# The effect of intensive exercise program and kinesiotaping following total knee arthroplasty on functional recovery of patients

**DOI:** 10.1186/s13018-018-0924-9

**Published:** 2018-09-12

**Authors:** Birhan Oktas, Ozge Vergili

**Affiliations:** 1 Orthopaedie and Traumatology Department, Kirikkale University Faculty of Medicine, Yurtkur Sokak, No:1 Yahsihan, Kirikkale, Turkey; 2 Physiotherapy and Rehabilitation Department, Kirikkale University Faculty of Health Sciences, Kirikkale, Turkey

## Abstract

**Background:**

Total knee arthroplasty (TKA) is a gold standard for terminal term gonarthrosis patients in order to diminish pain, correct deformities, and regain stability. Postoperative functional recovery of patients depends on the current postoperative rehabilitation program to an important extent. The purpose of our study is to compare midterm functional level of gonarthrosis patients who were included in physiotherapy rehabilitation program following TKA with people in similar ages without any surgical indication nor intervention. We have aimed also to compare functionality of people who had additional kinesiotaping (KT) treatment with people who had conservative treatment only after knee arthroplasty.

**Methods:**

Functional level and knee functionality of people in study were evaluated at the end of postoperative first month by 6-min walk test and Lysholm knee score respectively.

**Results:**

There was no statistical difference between groups with and without TKA in terms of height, weight, body mass index, Lysholm score, and 6-min walk test score (*p* > 0.05). In patient group with TKA, people with postoperative KT treatment had greater 6-min walk test score for postoperative first month (*p* = 0.005).

**Conclusion:**

TKA is considered a gold standard procedure in order to increase the quality of life and improve functionality of terminal term gonarthrosis patients. It is necessary to immediately control pain and edema of knee and apply intensive rehabilitation program aimed at muscle strengthening around hip and knee in order to increase the success of operation.

## Background

The knee joint is one of the major joints that take an important place in the functionality of a person in everyday life. Therefore, in the advanced stages of gonarthrosis, the quality of life of an individual considerably decreases. In this direction, total knee arthroplasty (TKA) is used as the gold standard in coping with pain, deformity, and instability, especially in terminal term gonarthrosis patients [1–3]. The reason for the fact that TKA is a procedure that can be accepted as the gold standard is that postoperative results are quite satisfactory [4]. However, the share of physiotherapy and rehabilitation programs applied only in the postoperative period is rather big in these results in cases in which they are initiated in the preoperative period and continued for a certain time in the postoperative period, and the patient cannot be reached in the preoperative period because it is not possible for the surgery alone to bring especially the lower extremity muscle strength to the level of a healthy population [5]. In patients in the advanced stages of gonarthrosis, the decrease in the lower extremity muscle strength already reaches a certain level in the preoperative period and progresses further in the postoperative period [6–8]. Thus, the rehabilitation of the knee joint, of which stabilization is ensured primarily by soft tissues, plays an important role in the improvement of the functional levels of patients. The place of exercise programs in rehabilitation is undoubtedly great. Exercise programs can be applied under the supervision and control of supervisor physiotherapists, as well as in the form of in-home programs, provided that they are taught to the patient before discharge and are revised and updated periodically [9–11]. One of the most important factors affecting the success of both surgery and preoperative rehabilitation programs is edema and pain around the knee in the early postoperative period. Edema and pain cause inadequacies in the functional capacity of the individual in the early and late postoperative period [12, 13]. Therefore, treatment modalities for edema and pain in the early postoperative rehabilitation should not be ignored. In this respect, kinesiotaping (KT) treatment, which is practical, comfortable, and safe for the patient, is one of the complementary treatment modalities with positive clinical results [14, 15]. KT, a treatment method developed by Kenzo Kaze [16], is used clinically for the control of pain and edema and improving motor function, especially in musculoskeletal disorders and injuries [17].

The aim of this study is to compare the midterm functional levels of gonarthrosis patients, who have been applied TKA and admitted to the physiotherapy and rehabilitation program in the postoperative period, with the individuals in the similar age group who do not have surgical indications and to perform a functional comparison of the cases additionally receiving anti-edematous KT treatment in the group included in the rehabilitation program with the cases receiving only conservative treatment.

## Methods

### Cases

Twenty-four patients (study group) in the age range of 52–75 years who were diagnosed with stage 4 gonarthrosis according to the Kellgren-Lawrence radiological classification and underwent unilateral cemented TKA with the medial parapatellar retinacular approach and 17 patients (control group) with no surgical indication according to this classification were included in the study. The mean age of the study and control groups was 63.3 ± 6.9 and 63.6 ± 5.2 years, the body mass indices were 28.9 ± 4.0 and 29.7 ± 4.5 kg/m^2^, and the groups included two and one male patients, respectively. The exclusion criteria for the control group were as follows: having a value of 4 and above when the activity pain of the knee joints was questioned by a 10-cm visual analogue scale and having various lower extremity and/or back problems (fracture history, knee tumors, disc herniation, etc.) and cognitive impairment. The exclusion criteria for the study group were as follows: having a value of 4 and above when the activity pain of the non-operated knee joint was questioned by a 10-cm visual analogue scale, having bilateral arthroplasty, revision arthroplasty, various problems (fracture history, knee tumors, disc herniation, etc.) of operated and/or non-operated leg and/or back, developing complications secondary to the operation (deep vein thrombosis, infection, etc.), and having cognitive impairment. The study protocol was approved by Kırıkkale University, Ethics Committee for Clinical Trials, and an informed voluntary consent form was obtained from all the individuals participating in the study.

### Study design

Patients who had undergone TKA were divided into two groups using the random number generator. While conservative postoperative physiotherapy program was applied to one of the groups, KT treatment was applied to the other group during a 1-week hospital stay in addition to this program. The conservative exercise program was initiated on the second postoperative day. The exercise program performed by being combined with a 20-min local cold application every 34 h a day was initiated with ankle pump exercises, deep breathing exercises, isometric knee and hip exercises, sitting on the edge of the bed, and short-term walking with a load on the prosthetic side at a tolerable level; the number and frequency of the exercises were increased; the isotonic knee and hip circumference strengthening exercises were performed; the program was advanced by increasing the sitting and walking distances; and finally, the patients were discharged at the end of the first week by giving ambulation training and a home exercise program. Stretching exercises were also addressed in the home exercise program, and patients were informed about the conditions and activities to be considered. In addition to this program, the lymphatic correction technique was applied to the parapatellar region using a 0–10% stretch to reduce edema and pain from the second postoperative day in the group receiving KT treatment. As the edema was in acute phase, EDF (epidermis, dermis, fascia) technique was used by cutting the tape into as much pieces as possible in order to direct the lymphatic correction effect of tape to the superficial subcutaneous tissues predominantly.

The tapes were changed every other day, and the new ones were applied. The patients were taped for the last time before being discharged and were asked to remove the tapes 3 days after as they had been taught.

### Outcome measures

The functional levels and knee functionalities of all cases included in the study were evaluated using the 6-min walk test and the Lysholm knee score, respectively. These evaluations of the TKA patients included in the study group were performed at the end of the first postoperative month. The 6-min walk test, which is an excellent tool for estimating functional ambulance [18], is frequently used clinically to evaluate the walking speed and endurance following TKA [19–21]. The 6-minute walk test, which is a reliable test for evaluating functionality in the patient population in question [22], is a highly reliable test that can also be used to evaluate the functional performance of patients following total hip arthroplasty [23]. The Lysholm knee scale evaluates pain, instability, locking, swelling, limping, stair climbing, crouching, and walking support need in the knee joint under eight main headings, and the Turkish version of the scale in question is a clinically feasible, valid, and reliable evaluation tool that can be used to evaluate the knee functionality in a variety of disorders of the knee joint [24] (Table 1).

**Table 1:** Lysholm knee score

Limping (5 points)	
Never = 5	
Mild or periodically = 3	
Strong and continuous = 0	
Support (5 points)	
No support = 5	
Walking stick or crutches = 2	
Impossible = 0	
Restraining (15 points)	
No restraining or restraining feeling = 15	
Has the feeling, but no restraining = 10	
Occasional restraining = 6	
Frequent = 2	
Joint restrained at examination = 0	
Instability (25 points)	
Never miss a step = 25	
Seldom, during athletic activities or other strong-effort exercises = 20	
Frequently during athletic activities or other strong-effort exercises (or unable to participate) = 15	
Occasionally in daily activities = 10	
Frequently in daily activities = 5	
At each step = 0	
Pain (25 points)	
No pain = 25	
Intermittent or mild during strong-effort exercises = 20	
Marked during strong-effort exercises = 15	
Marked during or after walking more than 2 km = 10	
Marked during or after walking less than 2 km = 5	
Continuous = 0	
Swelling (10 points)	
No swelling = 10	
Upon strong-effort exercises = 6	
Upon usual exercises = 2	
Continuous = 0	
Climbing stairs (10 points)	
No problem = 10	
Slightly damaged = 6	
One step at a time = 2	
Impossible = 0	
Squatting (5 points)	
No problem = 5	
Slightly damaged = 4	
Not exceeding 90° = 2	
Impossible = 0	
Total score: ________	

### Statistical analysis

Statistical analyses were performed using IBM SPSS Statistics 21 software. As descriptive statistics, number and percentage were used for qualitative data, and mean ± standard deviation were used for numerical variables. Whether numerical variables conform with normal distribution was examined by the Shapiro-Wilk test. Whether there was a difference in terms of the qualitative variable (gender) between the two groups was examined by Fisher’s exact test, and the difference in terms of numerical variables in the independent groups was analyzed by the *t* test. The *p* < 0.05 value was accepted to be statistically significant.

## Results

When the groups undergoing and not undergoing TKA were considered, no difference was found between the two groups in terms of age (*t* = − 0.123, *p* = 0.903). The gender distribution of the groups is not different (*p* = 1.000). There was no significant difference between the two groups in terms of body mass index (BMI), Lysholm score, and 6-min walk test score (*p* > 0.05) (Table 2). In the patient group which had undergone TKA, a difference was found in the 6-min walk test scores of individuals receiving and not receiving KT treatment only in the postoperative first month (*p* = 0.005) (Table 3).

**Table 2:** Comparison of the patients according to applied treatment (surgery or not)

Variables	Case (*n* = 18)	Control (*n* = 17)	Test statistics	*p*
Age (years)	63.3 ± 6.9	63.6 ± 5.2	− 0.123	0.903
Gender M/F	2/18	1/17		1.000
Body mass index (kg/m^2^)	28.9 ± 4	29.7 ± 4.5	− 0.525	0.603
Lysholm score	87.5 ± 8.6	86.2 ± 10.2	0.398	0.693
6-min walk test score (meter)	364.8 ± 63.4	325.2 ± 73.8	1.704	0.098

The comparison of the two groups undergoing and not undergoing TKA in terms of the age, gender, height, weight, body mass index (BMI), Lysholm score, and 6-min walk test score

**Table 3:** Comparison of the patients according to kinesiotaping (KT) application

Variables	KT available	KT not available	*Z*	*p*
Gender (M/F)	2/10	0/6		0.529
Age	61 (54–75)	67 (53–74)	− 0.703	0.482
Body mass index	30.6 (24.3–36)	24.973 (23.4–34.4)	− 2.154	*0.031*
Lysholm score	90.5 (76–99)	89.5 (70–99)	− 0.330	0.741
6-min walk test score	408 (280–460)	307.5 (250–345)	− 2.777	*0.005*

The comparison of the patients receiving and not receiving KT treatment in the TKA-applied group in terms of the age, gender, height, weight, body mass index (BMI), Lysholm score, and 6-min walk test score. Only the differences between two groups are statistically significant in terms of BMI and 6-min walk test score

## Discussion

Gonarthrosis is the most common joint disease, in which stopping the degenerative process is not possible. The gold standard for diminishing pain, correcting deformities, and regaining stability in terminal term patients is total knee arthroplasty (TKA) [1–3]. Following TKA, especially at the end of the second year, it has been shown that the cardiovascular compliance level of patients increases in parallel with their functionality. Thus, the TKA approach has been shown to have a positive effect on the quality and length of patients’ life [25].

In patients with end-stage symptomatic knee osteoarthritis (OA), while TKA improves the physical function based on a personal report [26], a performance-based physical function such as walking speed improves more slowly. Walking speed limitations can last even for a year or more after the surgery [27]. In a study, by Pua et al., investigating factors affecting walking speed after acute post-TKA, it was determined that the quadriceps femoris muscle strength and range of knee joint motion are important physical factors affecting walking speed, especially on the contralateral side, and fear-based cognitive factors, which result in activity limitation, also affect this physical parameter [28]. Similarly Christensen et al. underlined the importance of quadriceps muscle strength on gait impairments following TKA [29].

There are some differences in the recovery process of patients following TKA. Most of the patients experience functional deficits in the lower extremity muscles, such as weakness and slower walking speed, difficulty in climbing up and down the stairs, and standing up from a chair, compared with healthy individuals of the same age [30–32]. Therefore, it is necessary to consider other joints, especially the hip joint as an underlying cause of this functional impairment in the patient group in question. It is once again confirmed that the hip joint has to be examined considering the hip muscle strength and hip joint mechanics required for normal knee joint functioning.

The weakness in the hip abductor muscles observed in knee OA patients may persist following TKA [33]. The strength of this muscle group in post-prosthetic patients has an important effect on the faster recovery of the knee function [34]. Therefore, it will be meaningful that exercise protocols for strengthening hip muscles take place in the routine rehabilitation programs following TKA [35]. Piva et al. emphasized the importance of the hip circumference muscle strength for the functional recovery following TKA. It was shown that the strength of the hip abductor muscles following TKA affects the physical function of patients more than the demographic or anthropometric measures or the quadriceps muscle strength.

It is known that one of each three TKA patients has residual knee pain at the end of the second postoperative year and the functional capacity of the knee has inadequacies [36]. In a study, carried out by Mizner et al. to identify the time course of the restoration of inadequacies and function following TKA and which was based on the hypothesis that the correlation between the functional performance and quadriceps muscle strength is stronger than knee flexion ROM or pain, patients were evaluated in the first, second, third, and sixth months following the 6-week physiotherapy program after discharge. Patients recovered faster than expected, and long-term outcomes were found to be better than previously reported in the literature. The high correlation between the quadriceps strength and functional performance once again emphasized the importance of muscle-based approaches in the postoperative rehabilitation programs.

The recovery of the muscle strength around the knee following TKA is very difficult. The strength deficit in the quadriceps muscle can continue at the rate of 30% even at the end of the second postoperative year. A similar level of postoperative deficits in the hamstring muscle strength can also continue for years [37]. An increase is observed in the quadriceps/hamstring co-activation due to muscle deficits in the muscle groups in question. This increase disrupts the walking kinetics and kinematics and load distribution mechanism of the knee joint and results in the continuity of the degenerative process in the knee joint [38–40]. Therefore, bilateral strength and muscle activation training should be initiated in the earliest period following TKA in order to optimize the muscle firing patterns. The strength of the knee extensor muscles is very important for the patient satisfaction and functional activity restoration following TKA; therefore, quadriceps strengthening exercises should be focused on postoperative rehabilitation programs [41]. Especially that the hamstring muscle group is skipped in rehabilitation programs, this results in a residual functional deficit of patients. In order to optimize the muscle function and long-term rehabilitation outcomes, the improvement of the hamstring muscle strength should be emphasized as much as quadriceps in the rehabilitation program following TKA [42].

In a study conducted by Artz et al., it was shown that conventional physiotherapeutic exercise approaches performed after primary total knee replacement provided short-term improvements in the physical function, but these gains were not long term [43]. It was reported that more intensive therapeutic approaches were required for patients to achieve functionality following TKA at the level of healthy cases and that standard rehabilitation programs only allow patients to return to their functional levels in the preoperative period [44].

Merk et al. conducted a study on rehabilitation programs following TKA; it was emphasized that sports-specific therapy was required following the conventional rehabilitation program in order to prolong the functional life of the prosthesis and to prevent falls and inappropriate mechanical stress on the joint and that weakness in the quadriceps muscle group would persist with the application of only conventional rehabilitation [45]. Neuromuscular electrical stimulation and higher intensity strength training programs should be considered in addition to the traditional rehabilitation approaches for accelerating the functional recovery of patients following TKA. In cases when optimal results are still not achieved, aquatic exercises and eccentric strengthening training should be focused on [46]. Bade and Stevens-Lapsley showed in their clinical study that a high-intensity rehabilitation program following TKA improves both short-term and long-term functionality and quadriceps muscle strength compared to a low-intensity one, and the high-intensity program does not cause any complications [47].

Postoperative edema and pain are important factors affecting the success of rehabilitation. It has been concluded that decreased knee extension strength, which leads to a decline in the functional performance following TKA, may be due to postoperative edema around the knee, and the edema control in the early postoperative period in rehabilitation protocols will positively affect the success of rehabilitation and the early functionality level [48].

In the acute postoperative period following TKA, both cold and kinesiotaping applications reduce pain and swelling, thus increasing the functional performance. Yüksel et al. showed that although these modalities have no advantage over each other, they have no acute effects on the muscle strength [49].

Another study by Donec et al. showed that the KT applied within the scope of an early rehabilitation program following TKA reduced postoperative pain and edema and increased the range of motion of the knee extension [50].

Deniz et al. found KT application to be an effective rehabilitative approach for relieving pain and improving function in the acute stage of TKA as they found manual lymphatic drainage to be [51].

In the musculoskeletal problems of lower extremities, KT was found to have positive effects on pain, edema, and specific venous symptoms, but no evidence was found for its effects on the muscle strength, health-related quality of life, and joint function [52].

## Conclusion

In the present study conducted in the light of this information, it was found out that the rehabilitation program, which we initiated in the early postoperative period following TKA, improved the functional recovery of patients in a positive way and that at the end of the first postoperative month, prosthetic patients were found to have similar knee functionalities with non-prosthetic patients. We think that in the obtainment of this outcome, the fact that the exercise program that we applied ensured the necessary concentric and eccentric loading on the hip circumference muscles, and also on the quadriceps and hamstring muscles, has a great share. Our findings were supported by Pozzi et al. who underlined the importance of strengthening exercises in rehabilitation program following TKA [53]. Again, with the cold application initiated immediately after the surgery, postoperative edema and pain were taken under control early, and reflex muscle inhibition due to surgery was prevented, and thus, the functional recovery of patients was accelerated by increasing compliance and participation in exercise programs. Pain and edema control was achieved in a shorter time in TKA patients who received KT treatment in addition to cold application, and these patients were found to have higher walking distances of the 6-min walk test in the first postoperative month than those who did not receive KT treatment. Thus, the necessity of the inclusion of KT in the clinic following TKA from the early postoperative period has been revealed once again.

### Limitations of the study

The number of individuals per groups is not the ones desired by the researches. It can be attributed to wide range of exclusion criteria and patients’ voluntary basis nature of our study. Secondly, radiological evaluation of knees of individuals in the control group could have given a chance to understand the level of arthritic changes in their joint. Finally, knee scales in addition to Lysholm knee score could have been used to evaluate the functional status of individuals.

## Abbreviations

KT: Kinesiotaping
OA: Osteoarthritis
TKA: Total knee arthroplasty
